# Modelling the Transference of Trace Elements between Environmental Compartments in Abandoned Mining Areas

**DOI:** 10.3390/ijerph17145117

**Published:** 2020-07-15

**Authors:** Fernando Barrio-Parra, Miguel Izquierdo-Díaz, Luis Jesús Fernández-Gutiérrez del Álamo, Bárbara Biosca, Eduardo De Miguel

**Affiliations:** Prospecting & Environment Laboratory (PROMEDIAM), Universidad Politécnica de Madrid, 28003 Madrid, Spain; fernando.barrio@upm.es (F.B.-P.); miguel.izquierdo@upm.es (M.I.-D.); luis.fdezgda@upm.es (L.J.F.-G.d.Á.); barbara.biosca@upm.es (B.B.)

**Keywords:** cellular automata, soil, sediment, water, pollution, trace elements

## Abstract

An openly accessible cellular automaton has been developed to predict the preferential migration pathways of contaminants by surface runoff in abandoned mining areas. The site where the validation of the results of the Contaminant Mass Transfer Cellular Automaton (CMTCA) has been carried out is situated on the steep flank of a valley in the Spanish northwestern region of Asturias, at the foot of which there is a village with 400 inhabitants, bordered by a stream that flows into a larger river just outside the village. Soil samples were collected from the steep valley flank where the mine adits and spoil heaps are situated, at the foot of the valley, and in the village, including private orchards. Water and sediment samples were also collected from both surface water courses. The concentration of 12 elements, including those associated with the Cu-Co-Ni ore, were analyzed by ICP-OES (Perkin Elmer Optima 3300DV, Waltham, MA, USA) and ICP-MS (Perkin Elmer NexION 2000, Waltham, MA, USA). The spatial representation of the model’s results revealed that those areas most likely to be crossed by soil material coming from source zones according to the CMTCA exhibited higher pollution indexes than the rest. The model also predicted where the probabilities of soil mass transfer into the stream were highest. The accuracy of this prediction was corroborated by the results of trace element concentrations in stream sediments, which, for elements associated with the mineral paragenesis (i.e., Cu, Co, Ni, and also As), increased between five- and nine-fold downstream from the predicted main transfer point. Lastly, the river into which the stream discharges is also affected by the mobilization of mined materials, as evidenced by an increase of up to 700% (in the case of Cu), between dissolved concentrations of those same elements upstream and downstream of the confluence of the river and the stream.

## 1. Introduction

The mining of metallic mineral deposits and its associated processes cause harmful environmental effects worldwide, such as trace element pollution, soil and water acidification (Acid Mine Drainage—AMD), and, ultimately, damage to human health and ecosystems. These activities may also modify the geomorphology of the site, producing landscape and geological impacts, such as flooding, landslides, and erosion [1]. The mining waste produced (at least one ton of waste is generated per ton of ore extracted [2]) is usually enriched in toxic trace elements. Therefore, areas that have supported mining activities in the past are potential sources of severe contamination nowadays, and they may pose an unacceptable health risk to neighboring populations [3,4,5,6,7,8,9,10,11,12]. The layout of the mining material on the site and the climatic, geomorphological, and hydrogeological characteristics of the area determine the mobility of trace elements, both in solution and in particulate form [1,13,14], and, consequently, their potential effects on receiving soils, sediments, groundwater, and surface water [15,16,17,18,19,20,21]. Soils that have supported historical mining activities are often less developed and enriched with heavy metals, and there is a relationship between the distance to the waste piles and the evolution of the soils [1]. Surface courses receiving runoff and subsurface water enriched with trace elements from areas with mining wastes have shown variations in their environmental quality parameters, such as pH or toxic trace element content, up to distances of more than 5 km downstream of the source zone [22,23,24].

A large number of studies have examined the transfer of inorganic contaminants into the biosphere in areas where metal mining had taken place. In particular, there are several references to soil-vegetation uptake as a tool for bioremediation in areas that are affected by mining wastes (e.g., [25,26,27,28,29,30,31,32,33]). Similarly, publications on the geochemical aspects of the transfer from source areas to other environmental compartments (soil, surface water, and groundwater) abound [1,34,35]. However, there have been relatively few attempts to explain and predict, through the application of mathematical models, these transfer mechanisms. There are multiple examples of applications for the estimation of speciation and solubility of trace elements in water [36], as well as for their transport via surface water [37], but there is a lack of predictive models for the transfer of elemental contaminants through erosion and drag mechanisms from the sites of accumulation of mining wastes to receiving soils and surface waters. These processes of gravitational mobilization are especially relevant in areas with a steep geomorphology, either natural or resulting from historical mining activity, and where the climate is rainy and/or with a tendency to torrential rainfall. This is particularly relevant in a climate change framework where the frequency of extreme weather events is expected to increase [38,39,40,41] and the mobilization of toxic elements into soils and surface waters through these mechanisms could significantly rise.

For all of the above reasons, the objectives of this work were (a) to develop a model to predict the main transfer routes of contaminants from mining wastes in regions with predominant surface transport and (b) validate the model’s predictions with geochemical data of soil, surface water, and sediment matrices from a site affected by abandoned mining activities.

## 2. Materials and Methods

### 2.1. Study Area

**Figure 1:**
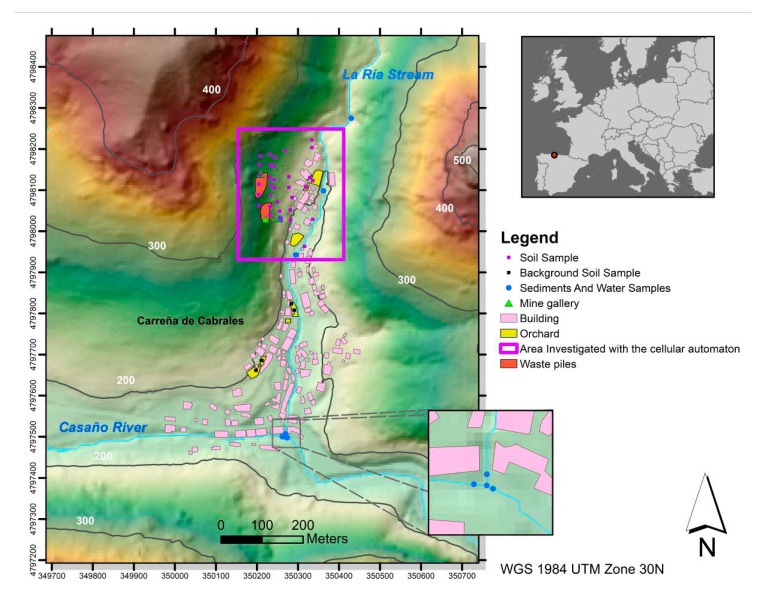
Location of the study area and position of sampling points.

The study site is located in the north of Spain, near the “Picos de Europa” National Park, and it includes a village with a population of 388 inhabitants [42], which is situated at the bottom of a valley, at an altitude of 200 m above sea level (m.a.s.l.). A stream runs through the valley, crossing the village from north to south and flowing into a larger river that circles the village to the south (Figure 1). On the western flank of the valley there are at least three mine galleries and two waste piles (between 220–260 m.a.s.l.) [43], associated with a former Co-Ni-Cu mine (Mina La Sierre). The mine, an example of eastern Asturian epithermal ores, started its activity in the mid-19th century and it was finally abandoned in the late 1960s [44]. The mineral paragenesis consists of chalcopyrite with small contents of pyrite, bravoite ((Ni Co, Fe)S_2_), and cobaltite (CoAsS), as well as other alteration minerals, such as erythrite (Co_3_(AsO_4_)_2_·8 H_2_O), annabergite (Ni_3_(AsO_4_)_2_·8 H_2_O), and heterogenite (CoO(OH)). Chalcocite (Cu_2_S), goethite (α-Fe^3+^O(OH)), lepidocrocyte (γ-Fe^3+^O(OH)), and malachite (Cu_2_CO_3_(OH)_2_) are also found associated with the mineral paragenesis at the site [45,46]. As a result of the historical mining operations the soils around the mined area are enriched in toxic elements, such as As, Ni, Pb, and Sb [45,46]. The slope of the terrain between the mine spoil heaps and the location of the potential receptors, i.e., human population (village inhabitants) and fluvial ecosystem (stream crossing the village), is approximately 50%. The climate at the site is characterized by abundant rainfall (792 mm/year) and mild temperatures (13.8 °C annual average) [47]. Occasional episodes of torrential rain occur in the study area, which, together with the steep slope, increase the transfer of soil from around the mine adits and spoil heaps to other environmental compartments, such as the orchards near homes and the stream, potentially causing an increase in the concentration of trace elements in both receiving bodies.

### 2.2. Sampling and Analysis

The field campaign involved the collection of 38 samples of soil along transects, starting at the height of the mine adits and waste piles and progressing down to the village, where soil of the village orchards was also sampled (four of those samples of orchard soil, collected outside the mine-influenced area, were used as background samples for the calculation of pollution indexes). Seven samples of water and riverbed sediment were collected along the stream and at its confluence with the river, and an additional water sample was taken from the mine adit (Figure 1). The number of samples collected of all three environmental matrices was lower than desirable, due to the slope and ruggedness of the terrain and the difficulties in accessing the water courses. This fact needs to be considered as a source of uncertainty when evaluating the validation of the results of the model.

Composite soil samples that were made up of three increments were collected with an Edelman auger from the top 20 cm of the soil profile. Water sampling took place the day after an episode of torrential rain (17 L/m^2^ [47]), which produced erosion on road embankments and small landslides in the vicinity of the study area, and allowed verifying whether the mass transfer phenomena caused by runoff that is considered in the cellular automaton had a measurable effect on the chemical properties of water courses in the study area. The water samples were filtered in situ to remove suspended solids and concentrated HNO_3_ was added to sample containers until a pH of 1.5–2 was reached to ensure proper preservation. At each water sampling point, sediment samples were also collected and sieved to 100 μm in-situ, and the main physicochemical parameters (conductivity, pH, temperature, and redox potential) were measured. The samples were kept refrigerated during transport to the laboratory where soils and sediments were dried at 105 °C until constant weight and subsequently digested with aqua regia (3 HCl:1 HNO_3_), following the ISO 11466:1995 experimental protocol. Quality controls included on-site collection of two soil samples in triplicate, method blanks, and a certified reference material (ISE sample 995 of Sandy Soil, WEPAL). The samples were analyzed by ICP-OES (Perkin Elmer Optima 3300DV, Waltham, MA, USA) and ICP-MS (Perkin Elmer NexION 2000, Waltham, MA, USA) for metal and metalloid concentrations. Coefficients of variation (CV) of analytical results were less than 5% for approximately 95% of the soil and sediment samples and 90% of water samples (only As in two soil samples, Co in one sediment sample, and Sb in five water samples exceeded a CV of 10%). Recovery factors relative to the reference material were all in the range of 88% to 112%. Coefficients of variation of the soil triplicates were all less than 6%, except for Sb in one of the them, with a value of 8%.

### 2.3. Modelling of Trace Element Mobility Between Environmental Compartments

A cellular automaton was developed to model the transfer of contaminants through surface run-off processes. Cellular automata are algorithms that allow for the study of the behavior of natural systems (discretized in cells) through rules of interaction between the elements that are part of the system. They have been used, for example, to model the geomorphological dynamics of dune systems [48,49,50]. Analogous to the avalanche module applied to dune dynamics, the model used in this study (Contaminant Mass Transfer Cellular Automaton: CMTCA) calculates the slopes around a cell evaluated in a Digital Elevation Model (DEM) to predict possible migration routes of rain-dragged soil into a stream.

Several studies have considered the application of cellular automata in the simulation of surface run-off processes [51,52,53]. In addition to employing neighborhood rules to estimate flow direction based on elevation differences, these models require the assessment of other parameters, such as infiltration and rainwater interception by vegetation. Unlike them, CMTCA is a probabilistic model that is designed to predict, with a low demand for input data, the probability of soil transport over a land surface from a defined source area (i.e., areas around spoil heaps with trace element-enriched soil) until a point of transfer to other environmental compartments (for example, surface water bodies).

The CMTCA model (available as Supplementary Materials) uses data matrices in ASCII format, with a maximum mesh size of 2000 × 2000 cells, which can be generated through Geographic Information Systems (GIS). The model’s input data are: (1) the DEM, (2) source areas (soils with high concentrations of trace elements, such as around mine waste piles or mine adits), (3) barriers (architectural elements that prevent surface water flow such as buildings and walls), and (4) sinks (surface water courses).

Starting with those cells defined as “source cells”, the algorithm calculates the slopes of the eight nearest neighbors, excluding cells classified as barriers and those with elevations that are equal to or greater than the central one. The model randomly selects one of them with a probability proportional to the calculated slope. The model repeats the analysis starting from the selected cell until a sink cell is reached. The algorithm is executed a high number of times (*n* = 1000) calculating the probability that water or soil from a source zone circulates over the cell. The result of the model can identify both the route followed by mobilized material enriched in trace elements and the point of discharge to the stream.

In this work, a DEM with a resolution of 5 m [43] was chosen, mine waste piles were selected as source areas, and buildings and orchards with impermeable fencing were defined as barriers. Source areas and barriers were delineated with the aid of digitalized orthophotos [54] and validated in the field. The layout of rivers and streams was obtained from vector layers of SITPA [55].

In order to evaluate the coherence between the predictions of the cellular automaton and the analytical results in soils, additive contamination indices (*PI_sum_*, Equation (1)) were calculated [56] and their spatial distribution was compared with the results of the cellular automaton. Soil concentrations (*C_i_* in Equation (1)) of Cd, Co Cr, Cu, Fe, Mn, Ni, Pb, and Zn were used in the calculation of the contamination indices. The average concentration of those elements in four samples from orchards, at a distance of more than 250 m to the south of the source areas (Figure 1), where there is no influence of the mining activities, were taken as background concentrations (*C_Background_*).

(1) $$PI_{sum}=\overset{n}{\underset{i=1}{\sum }}\frac{C_{i}}{C_{Background}}$$

The ability of the model to predict the surface mass transport of contaminants was assessed by means of a Singular Value Decomposition (SVD) Principal Component Analysis (PCA) [57]. Lastly, the automaton’s estimated transfer areas of contaminants between the soil and the water/sediment compartments were spatially represented and compared with the concentration of trace elements in water and sediment along the stream.

## 3. Results and Discussion

### 3.1. Geochemical Characterization

Table 1, Table 2 and Table 3 summarize the results of the geochemical characterization of the site. To contextualize the potential risk for human health at the site, these tables include the regional Reference Concentrations (regional SSLs [58]) and the percentage of samples that exceed them. The majority of soil samples presented concentrations of the elements of commercial interest (Cu, Co, and Ni) and of the rest of elements present in the mineral paragenesis (As and Sb) above their respective reference values (Table 1). When compared to the average soil concentrations reported by Álvarez et al. [44] for the same area, the results of this study are almost identical for Sb and Zn, nearly twice as high (although with very similar maximum values) for Pb, and approximately 30% lower for the rest of the elements available for comparison (As, Co, Cu, Mn, and Ni). The Pearson correlation matrix (Table 4) reveals a strong association between Co-Cu-As-Ni-Sb, all of them included in the mineral paragenesis of the deposit. The relationship found between Pb and Zn is indicative of a deposit of magmatic affinity with an epithermal-type hydrothermal mineralization (90–110 °C) [59].

The interpretation of the analytical results of water and sediment samples needs to consider that, in both cases, samples were collected from two different receptor media: river and stream. Measurements of pH in water samples were fairly constant around the average value of 7.76, except for the sample from the mine adit., where the pH reached 8.26. Similarly, oxidation-reduction potential (average value = 460 mV) only slightly increased in the stream as it passed the village and again in the river, after the confluence of both water courses. Specific conductance, in turn, increased from below 100 μS/cm upstream from the village to values of approximately 700 μS/cm at the stretch of the stream included in the area modelled by the CMTCA.

The water sample that was collected from one of the mine galleries (source zone) presented concentrations above the guideline values for ecosystem health [60] for all the elements, except Pb and Zn, which also had exceeded their respective soil reference values in one or more soil samples. However, in the stream and river, after the episode of torrential rain, guideline values for ecosystem health were only exceeded for Cu, Ni, and Zn. In the case of sediments (Table 3), in the absence of specific legislation in Spain, the Freshwater Sediment Screening Benchmarks established by the USEPA [61,62] were used as references. Benchmark concentrations were exceeded for all elements except Fe, both in the source zone and in at least one sample taken from the water courses (in the case of Cu, the reference concentration was exceeded in four samples). As opposed to water samples, which are indicative of the environmental instant after the episode of torrential rain, the values obtained in sediments are representative of an integrated accumulation process over time.

### 3.2. Model Results and Validation

**Figure 2:**
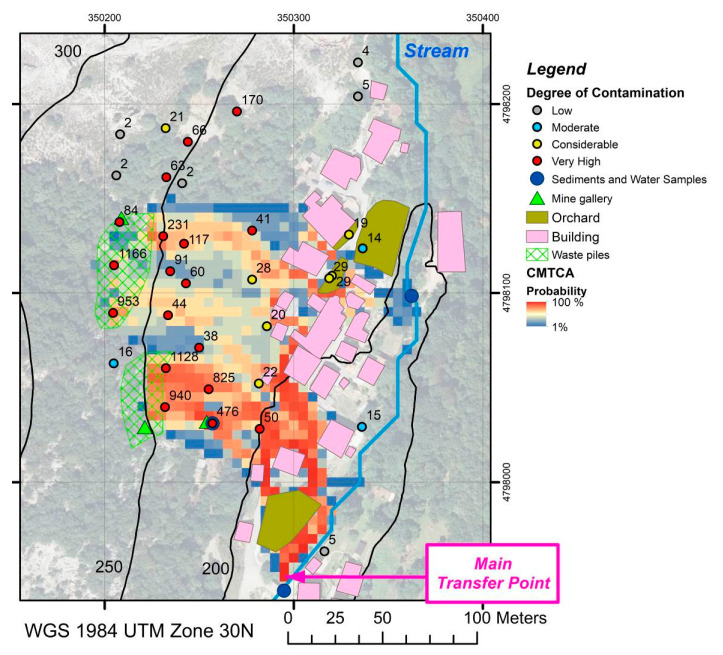
CMTA model results and pollution indices in soil samples.

The estimated pollution indices (*PI*_sum_) for soil samples, classified according to Qingjie et al. (2008) [56] in “low”, “moderate”, “considerable”, and “very high”, and the results obtained from the CMTCA model are shown together in Figure 2. Table 5 summarizes the background concentrations (*C_Background_*) to determine these pollution indices. Although one of the four background samples presented significantly higher contents of Cd and Cu than the rest, there were no evidences of contamination in the field or of cross contamination during transport, preparation and analysis and therefore the sample was retained for the calculation of background values. Background concentrations of the elements of commercial interest (i.e., Co, Cu, and Ni) were similar to their respective Q1 concentrations in the rest of the study area (Table 1), whereas elements that were unrelated to the mineral paragenesis presented background values very similar to their median concentrations (except Zn, whose average background concentration exceeded its Q3).

As expected, samples with the highest pollution indices (red dots in Figure 2) were those that were collected from source areas, i.e., mine waste piles (marked with a green frame in the map in Figure 2). The result of the model describes the gravitational transport of soil from those source areas, highlighting preferential flow pathways in reddish tones. Upon reaching the first buildings of the village at the foot of the valley flank, the east-bound mass flow of soil along the valley flank is diverted to the south, following the gentle slope in that direction of the village streets. As the flow progresses, the pollution indices decrease. Areas that are most likely to be crossed by soil coming from the source zones exhibit higher pollution indices (higher *PI_sum_*) than those with lower probabilities according to the model. It is worth remarking the model’s ability to exclude areas that cannot be affected by the deposition of contaminated soil: samples taken inside urban orchards isolated by an impermeable fence, samples taken along the river in the northern part of the study area, and samples collected at higher elevations than those corresponding to the mine waste piles.

Three points located to the north of the site presented PIs between 63 and 170, which did not agree with the results of the CMTCA model. The orthophoto layer of Figure 2 shows a change in vegetation cover from trees to bare rock in the northern sector of the study site. This discontinuity corresponds to a change in lithology, from limestone (tree-covered) to quartzite (bare rock). Points with PIs of 63 and 66 were still on the limestone, which is the rock that is mineralized, and not far from the historic mine workings. Even if there was no mining activity directly in the area where these two samples were collected, the host rock would continue to present high values of Cu-Co-Ni, which could explain the moderately elevated PI values found in them. The third sample with a PI of 170 was collected in an area where cartographic documentation indicates the existence of an old pile of gangue and low-grade ore [63]. Although the material accumulated there was removed, it is likely that it could have enriched the underlying soil, hence explaining the observed PI.

Table 6 summarizes the results of a principal component analysis (PCA) using “Singular value decomposition”, which analyzes the correlations between samples. The “loadings” that make up the main components (PC) help to interpret the relationships between the original geochemical variables and the two possible explanatory variables considered, i.e., CMTA: model output, and slope: of the terrain. The number of PCs considered has been set at three, since they explain a cumulative variance of more than 80% [57]. PC1 shows the relationship between concentrations of As-Co-Cu-Ni-Sb and the output of the CMTA model. This result is indicative of the association between the enrichment of these elements in the mine waste piles and the process of soil transport through an erosive and gravitational process described by the model output. Elements that are enriched in mining wastes are accumulated along the transport pathways predicted by the CMTCA model. PC2 correlates soil concentrations with the slope of the terrain in the study area. Elements that are associated with mining wastes present negative loadings in this component. This fact can be interpreted as a negative correlation between slope and concentration, a fact that reinforces the hypothesis of the gravitational transport process modelled with the CMTA, since areas with a lower slope favor the accumulation of material carried by surface runoff and, thus, result in higher concentrations of these elements in surface soil. Similarly to the elements that are associated with the mineralization, Pb and Zn in PC3 are inversely related to the slope (they are enriched in flat areas), but, unlike them, they do not show a strong relation with the CMTCA results, that is, they do not seem to follow the mass transport routes from the mine spoil heaps predicted by the model. Lastly, PC2 groups those elements that do not present a notable enrichment in the mineralization (Cd, Cr, Fe, Mn, V). Their positive, although not very strong, association with the slope is difficult to interpret. A possible explanation is that the concentration of these elements, which are naturally present in the native soil, becomes diluted with the material transported from the mine waste piles and accumulated in flat sections of the study area. In sections with a steeper slope, this accumulation and the subsequent dilution effect does not take place and the suite of elements grouped with positive loadings under PC2 recuperate their natural background levels.

**Figure 3:**
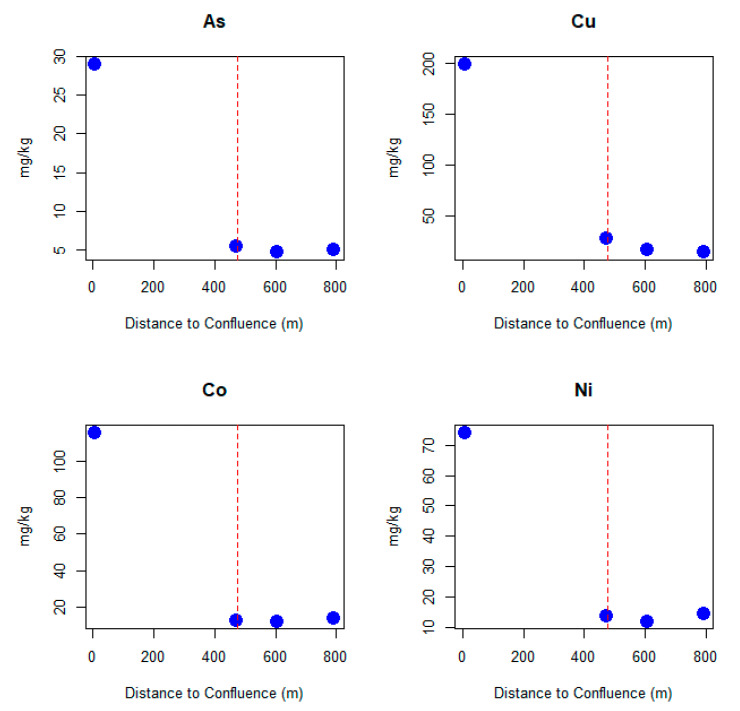
Distribution of trace element concentrations in stream sediment. Vertical dashed line indicates the main transfer point predicted by the CMTA model.

The application of the CMTA model also allowed to identify the most probable points of transfer of soil particles to the stream. The probability of this mass transfer is very low (blue colors) in the northern sector of the study area, null while the stream flows parallelly to the village, and very high (red colors) right after the southern end of the village (main transfer point), as illustrated in Figure 2. The evolution of the concentration of trace elements associated with the mined ore in the sediments of the stream (Figure 3) was used to validate the prediction of the CMTCA model. The concentration of As, Cu, Co, and Ni remains approximately constant, or only slightly increases, from the first sample, collected upstream from the village, until the third sample that is located right before the main transfer area predicted by the CMTCA model. After the main transfer area, concentrations in stream sediments rise significantly, i.e., between five-fold for As and more than nine times for Co, a fact that seems to corroborate the accuracy of the model’s predictions.

**Figure 4:**
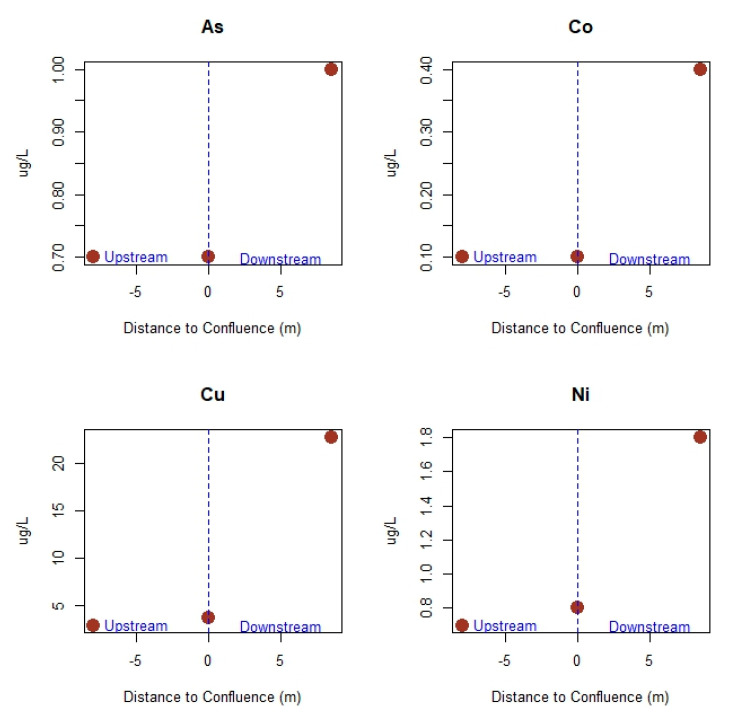
Examples of distribution of trace element concentrations in the river upstream and downstream of the stream discharge point.

Lastly, the effect of the mobilization of trace elements from the mine adits and mine spoil heaps down the slope of the valley flank and into the stream is further detected in the river into which the stream discharges, whose dissolved concentrations rise between 37% for As and up to almost 700% for Cu after the confluence of both water courses (Figure 4).

## 4. Conclusions

The predictions of the Cellular Automaton developed in this study (Contaminated Mass Transfer Cellular Automaton, CMTCA) have been validated with geochemical data (soil, surface water, and stream sediment) from a historical mining area in northern Spain. The model accurately predicts the most probable mobilization pathways followed by trace elements from source zones (mine adits and waste piles) down to the receiving stream, as evidenced by the fact that the highest pollution indices that were determined at the site were associated with samples collected along those pathways. It also successfully predicts the location along the stream banks where contaminant mass transfer to the stream is most likely to occur: Concentrations of trace elements in stream sediment remain relatively constant along the stream until they reach the predicted main transfer point, downstream from which they increase up to nine-fold for elements that are associated with the mined ore.

Because the CMTCA model has been proven to effectively delineate the spatial patterns of contaminant transport and mass transfer between environmental compartments, it could become a useful and inexpensive tool for the preliminary characterization of sites where surface mobilization of contaminated soil particles is an important transport mode of contaminants. In that same regard, it could also help to prioritize the allocation of investigation resources and direct them to the most highly impacted areas of the affected site. Lastly, the CMTCA could serve as the initial stage of more exhaustive models for the quantitative assessment of mass transfer rates in historical mining watersheds.

## Figures and Tables

**Table 1:** Summary of major and trace elements concentrations found in soil samples (*n* = 38) from the study area (mg/kg).

Element	Min	Q1	Median	Mean	Q3	Max	Reference Concentration [58]	% of Samples above Reference Concentration
**As**	7.23	24.7	107	418	339	3190 *	100	53
**Cd**	0.01	0.43	1.24	20.9	1.8	188	10	18
**Co**	2.54	46.2	239	1520	783	10,700 *	35	76
**Cr (total)**	1.02	7.32	13.7	226	24.3	4160	2 ^1^–10,000 ^2^	95 ^1^–0 ^2^
**Cu**	23.3	96.3	352	1690	1130	14,200 *	55	84
**Fe**	3000	12,300	15,800	19,400	25,900	41,100 *	NA **	NA **
**Mn**	15.6	353	1040	1550	2440	5160	6435	0
**Ni**	3.3	32.5	90	842	480	6270	65	63
**Pb**	8.92	30.4	50.7	53.7	72.3	122	70	26
**Sb**	1.25	5.22	9.43	16	18.6	73.7 *	5	74
**V**	7.33	13.7	20.8	28.9	44	59.2	100	0
**Zn**	10.8	59.6	87.2	113	143	294	455	0

* Sample collected from mine adit; ** Not Available; ^1^ Cr (VI); ^2^ Cr (III).

**Table 2:** Summary of major and trace elements concentrations (*n* = 8) in water samples (μg/L). Reference Concentrations: Minimum values that can cause damage from chronic exposure to any aquatic organism [60].

Element	Min	Q1	Median	Mean	Q3	Max	Reference Concentration ^1^	% of Samples above Reference Concentration
**As**	0.7	0.7	0.7	6.69	0.93	48.1 *	48 ^2^	12.5
**Cd**	<LD **	0.07	0.1	0.38	0.1	2.50 *	1.1	12.5
**Co**	0.1	0.1	0.25	46.2	0.48	368 *	5.1	12.5
**Cr (total)**	0.2	0.3	0.5	0.71	0.75	2.3	2 ^3^	12.5
**Cu**	1.8	3.5	10.7	23	32.8	68.3 *	0.23	100
**Fe**	48.8	64.2	94.6	114	138	269	158	12.5
**Mn**	1.4	2	2.3	2.7	3.33	4.7	1100	0
**Ni**	0.7	0.8	2.05	21.8	5.35	152 *	5	25
**Pb**	1	1.48	1.7	2.4	2.43	5.80 *	12.26	0
**Sb**	0.1	0.2	0.25	0.6	0.65	2.30 *	610	0
**V**	0.2	0.3	0.35	0.38	0.42	0.6	80	0
**Zn**	10.4	15.3	18.8	39.1	52.2	114 *	30	37.5

* Sample collected from mine adit; ** Limit of Detection; ^1^ Lowest Chronic Value for all organisms; ^2^ As (V); ^3^ Cr (VI).

**Table 3:** Summary of major and trace elements concentrations (*n* = 8) in sediment samples (mg/kg). Reference Concentrations: “Freshwater Sediment Screening Benchmarks (FSSB)” [61,62].

Element	Min	Q1	Median	Mean	Q3	Max	Reference Concentration	% of Samples above Reference Concentration
**As**	4.7	5.1	12.4	205	26.6	1170 *	9.8	37.5
**Cd**	0.4	0.41	0.49	0.61	0.7	1.16	0.99	12.5
**Co**	12	12.2	13.4	578	40.9	4430 *	50	25
**Cr (total)**	6.66	7.83	11.8	988	15.9	7820 *	43.4	12.5
**Cu**	14.3	25	67.1	3030	134	23,800 *	31.6	62,5
**Fe**	1220	10,600	14,000	12,800	16,400	19,100	20,000	0
**Mn**	343	395	655	917	1100	2620 *	460	50
**Ni**	12	14.2	20	426	35.5	2430 *	22.7	25
**Pb**	19.3	22.2	30.6	46.6	46.6	79.7	35.8	50
**Sb**	0.54	1.06	2.31	6.7	3.4	30.7 *	2	50
**V**	8.99	10.7	14	14	17.6	18.6	NA **	NA **
**Zn**	70.3	79.5	87.2	121	167	216	121	37.5

* Sample collected from mine adit; ** Not Available.

**Table 4:** Correlation matrix for concentrations in soil samples (*n* = 19). Statistical significance level code: ***—99.9%, **—99%, *—95%, and °—90%.

Element	As	Cd	Co	Cr	Cu	Fe	Mn	Ni	Pb	Sb	V	Zn
**As**	1		***		***	*		***	*	***		
**Cd**	0.2280	1		*		***	***		*		***	.
**Co**	0.9982	0.2315	1		***	*		***	*	***		
**Cr**	−0.1794	0.4851	−0.1943	1		.	*				**	
**Cu**	0.9931	0.2218	0.9964	−0.1770	1	*		***	*	***		
**Fe**	0.4609	0.8166	0.4678	0.3954	0.4823	1	***	*		*	***	
**Mn**	0.0081	0.8683	0.0082	0.5066	0.0044	0.7877	1				***	
**Ni**	0.9934	0.2066	0.9954	−0.1730	0.9986	0.4723	−0.0107	1	*	***		
**Pb**	0.5192	0.4962	0.5229	0.3678	0.5249	0.3253	0.2015	0.5122	1	**		***
**Sb**	0.9584	0.3650	0.9553	−0.1512	0.9458	0.5151	0.1174	0.9351	0.6028	1		
**V**	−0.0808	0.7098	−0.0911	0.6350	−0.0779	0.7813	0.8399	−0.0829	0.0038	0.0063	1	
**Zn**	0.2177	0.4404	0.2281	0.3228	0.2348	0.1448	0.1905	0.2257	0.8082	0.2710	−0.1122	1

**Table 5:** Analytical results (*n* = 4) obtained in unaffected urban orchards (used as background concentrations for the calculation of pollution indexes) (mg/kg).

Statistical Parameter	Cd	Co	Cr	Cu	Fe	Mn	Ni	Pb	Zn
**Mean**	0.44	24.2	14.0	92.5		1700	26.8	53.4	174
**SD**	0.66	12.9	4.07	52.7	2100	715	12.8	16.6	44.2

**Table 6:** Results (loadings) of the Principal Component Analysis.

Variable	PC1	PC2	PC3
**As**	0.36	−0.17	0.08
**Cd**	0.21	0.38	−0.14
**Co**	0.36	−0.17	0.08
**Cr**	0.02	0.36	−0.29
**Cu**	0.36	−0.17	0.07
**Fe**	0.28	0.33	0.12
**Mn**	0.13	0.45	−0.02
**Ni**	0.36	−0.17	0.08
**Pb**	0.25	0.00	−0.48
**Sb**	0.37	−0.12	0.04
**V**	0.08	0.47	0.14
**Zn**	0.14	0.02	−0.59
**CMTA**	0.33	0.02	0.19
**Slope**	0.02	0.26	0.48
**% Cumulative Explained Variability**	47%	74%	90%

## Supplementary Materials

The following are available online at https://www.mdpi.com/1660-4601/17/14/5117/s1: Contaminant Mass Transfer Cellular Automaton (CMTCA) Software.
