# Supplementary material for: Modelling the Transference of Trace Elements between Environmental Compartments in Abandoned Mining Areas
Source: Int J Environ Res Public Health. 2020 Jul 15;17(14):5117. doi: 10.3390/ijerph17145117 (PMC7400299; doi:10.3390/ijerph17145117)
Supplement: Supplementary file 1 [file ijerph-17-05117-s001.zip › CMTCA/Instructions.pdf]

Contaminant Mass Transfer Cellular Automaton: CMTCA: Instructions of use

Authors: Fernando Barrio-Parra and Luis Jesús Fernández-GutirrezdelAlamo

Installation

Launch the CMTCA.msi windows installer

Choose the folder where the program should be installed. A shortcut of the CMTCA model will be created in the desktop.

Use

1. Launch the CMTCA program.
2. Make sure the decimal separator in your files (periods, commas) matches the one selected in the "Regional and Language Options" in your computer settings.
3. Read the following ASCII matrices .txt files. The number of columns and rows, the X, Y corner coordinates, the cell size and the No Data value must be consistent between files.
    a. Digital Elevation Model (DEM)
    b. Source Areas: Cells within the source areas must be set to 1. All other cells should have a No Data value. Source areas pixels must not be adjacent between them.
    c. Streams: Cells included in streams must be set to 1. All other cells should have a No Data value.
    d. Barriers. Cells which act as barriers must be set to 1. All other cells should have a No Data value.
4. Enter the number of simulations (a numerical value between 1 and 2000).
5. Execute the model
6. Write the output ASCII file. Enter the root and the name of the file with the .txt extension.
